# Influence of temperature on thromboelastometry and platelet aggregation in cardiac arrest patients undergoing targeted temperature management

**DOI:** 10.1186/s13054-016-1302-9

**Published:** 2016-04-30

**Authors:** Anni Nørgaard Jeppesen, Hans Kirkegaard, Susanne Ilkjær, Anne Mette Hvas

**Affiliations:** Research Centre for Emergency Medicine, Aarhus University Hospital, Nørrebrogade 44, Building 30, 8000 Aarhus C, Denmark; Department of Anaesthesiology and Intensive Care Medicine, Aarhus University Hospital, Palle Juul-Jensens Boulevard 99, 8200 Aarhus N, Denmark; Center for Haemophilia and Thrombosis, Department of Clinical Biochemistry, Aarhus University Hospital, Palle Juul-Jensens Boulevard 99, 8200 Aarhus N, Denmark

**Keywords:** Coagulation, Heart arrest, Haemostasis, Hypothermia, Platelet function test, Thromboelastometry

## Abstract

**Background:**

Coagulation can be visualised using whole blood coagulation analyses such as thromboelastometry and platelet aggregation tests; however, the role of temperature in the analyses is ambiguous. The aim was to examine whether temperature influences the whole blood coagulation tests.

**Methods:**

We included 40 patients treated with targeted temperature management (33 ± 1 °C) after out-of-hospital cardiac arrest. The blood samples were obtained on hypothermia and normothermia. Each blood sample was analysed simultaneously at 33 °C and 37 °C by thromboelastography (ROTEM®) employing the assays EXTEM®, INTEM®, FIBTEM® and HEPTEM®, and by Multiplate®Analyzer, using COLtest®, ADPtest®, ASPItest® and TRAPtest® as agonists. Data on antithrombotic drugs were collected systematically from medical records, and data were analysed using repeated measurement analysis of variance (ANOVA).

**Results:**

The ROTEM® analyses showed increased clotting time, lower maximum velocity and increased time to maximum velocity (all *p* values <0.02) when performed at 33 °C compared with 37 °C, irrespective of the patients being hypothermic (median 33.1 °C) or normothermic (median 37.5 °C). However, EXTEM® time to maximum velocity showed no difference between the analyses performed at 33 °C and 37 °C when the patients were hypothermic (*p* = 0.83). No differences were found in maximum clot firmness (all *p* values >0.09) analysed at 33 °C and 37 °C, independent of the body temperature.

In the hypothermic blood sample, no difference was found when using the COLtest®, ASPItest® or TRAPtest® to compare platelet aggregation analysed at 33 °C and 37 °C (all *p* values >0.19), but platelet aggregation was significantly higher using the ADPtest® (*p* < 0.001) when analysed at 33 °C. In the normothermic blood sample, the TRAPtest® showed no difference (*p* = 0.73) when performed at 33 °C; however, significantly lower aggregation was found using the COLtest® and ASPItest® (all *p* values <0.001), while a higher aggregation at 33 °C was found using the ADPtest® (*p* = 0.003).

**Conclusion:**

ROTEM® analyses seemed not to be dependent on body temperature but showed a slower initiation of coagulation when analysed at 33 °C compared with 37 °C. The Multiplate®Analyzer results were dependent on the temperature used in the analyses and the body temperature. In whole blood coagulation tests, the temperature used in the analyses should be kept at 37 °C irrespective of the patient’s body temperature being 33 °C or 37 °C.

## Background

Severe cerebral damage is frequently observed in survivors of cardiac arrest [1]. However, the extent of the damage may be reduced by treating the patients with targeted temperature management [2, 3]. Evidence from trauma and surgical patients shows that unintended hypothermia is associated with an increased risk of bleeding [4–11]. Thus, it appears that targeted temperature management impairs haemostasis.

The dynamic coagulation process can be visualised by thromboelastometry (ROTEM®/TEG®), which is used in bleeding patients [12]. Platelet aggregation can be evaluated by several methods, but assessment by impedance aggregometry employing the Multiplate®Analyzer [13] is increasingly common. Analyses involving both thromboelastometry and Multiplate®Analyzer are performed at 37 °C, irrespective of the patient’s body core temperature. This implies that impaired haemostasis may be overlooked in hypothermic patients.

We hypothesised that whole blood coagulation, evaluated by ROTEM® and Multiplate®Analyzer, would reveal impaired haemostasis in patients treated with targeted temperature management after cardiac arrest, analysed at 33 °C instead of 37 °C. Our aim was to examine ROTEM® and Multiplate®Analyzer measurements analysed simultaneously at 33 °C and 37 °C in patients treated with targeted temperature management after cardiac arrest.

## Methods

This study was a prospective cohort study conducted from March 2013 to March 2014 at the Intensive Care Unit of Aarhus University Hospital, Denmark. It is a sub-study of the trial entitled “Time-differentiated Therapeutic Hypothermia” (ClinicalTrials.gov Identifier: NCT01689077). In this study, comatose patients resuscitated after cardiac arrest were randomised to either 24 hours or 48 hours of target temperature management (33 ± 1 °C). Inclusion criteria were as follows: return of spontaneous circulation (ROSC) after out-of-hospital cardiac arrest of presumed cardiac cause, Glasgow Coma Score <8, and age ≥18 years and <80 years. The exclusion criteria were the following: >60 minutes from circulatory collapse to ROSC, time interval >4 hours from cardiac arrest to initiation of target temperature management, terminal illness, coagulation disorder, unwitnessed asystolia, cerebral performance category 3–4 before the cardiac arrest, pregnancy, persistent cardiogenic shock (systolic blood pressure <80 despite inotropic treatment), new apoplexy or intracerebral haemorrhage, or lack of consent. Written informed consent was obtained from the relatives and from the patients themselves if they became capable of signing informed consent. The Danish Data Protection Agency and the Central Denmark Region Committees on Health Research Ethics approved the study (case number 20110022), and the study was performed in accordance with the Helsinki Declaration.

We included 40 patients resuscitated after out-of-hospital cardiac arrest, 5 of whom have previously been described by Nielsen et al. [14]. A medical doctor arriving by ambulance in a pre-hospital setting treated the patients prior to arrival at hospital. The pre-hospital protocol instructed the doctor to initiate cooling using intravenous NaCl (30 ml/kg) at 4 °C. All patients were treated with targeted temperature management for either 24 hours or 48 hours using surface cooling or intravascular cooling, and the patients were subsequently rewarmed at a rate of 0.5 °C/hour. Sedation during targeted temperature management was achieved by intravenous administration of remifentanil and propofol.

The body core temperature upon admission was measured in the ear or in the bladder, whereas the body core temperature at the intensive care unit was measured continuously in the bladder. If the patient had been discharged to the ward at the time of the normothermic blood sample, the body core temperature was measured as rectal temperature.

Information about the use of antithrombotic drugs was collected from the patients’ medical records. “No ADP inhibitors” referred to patients with no use of adenosine diphosphate receptor inhibitors (ADP inhibitors) at any time points, and “with ADP inhibitors” referred to patients who used APD inhibitors 24 hours prior to obtaining the blood sample. A similar distinction was made in relation to the use of aspirin; “no aspirin” meaning no aspirin treatment at any time point and “with aspirin” meaning treatment 24 hours prior to obtaining the blood sample.

### Blood samples

The first blood sample was obtained upon admission to hospital, the second after 22 ± 2 hours of targeted temperature management (the hypothermic blood sample), and the third at normothermia 48 ± 2 hours later (the normothermic blood sample). Blood samples were drawn from an arterial line or collected from the cubital vein or a central venous catheter using a 21G needle. At hypothermia and normothermia, citrate (3.2 %) blood collecting tubes were used for thromboelastometry (ROTEM®, Tem International GmbH, Munich, Germany), employing the assays EXTEM®, INTEM®, FIBTEM® and HEPTEM® (Tem International GmbH, Munich, Germany), coefficient of variation for all parameters <15 %. Hirudine anticoagulant tubes were used for platelet aggregation tests (Multiplate®Analyzer, Roche, Diagnostics GmbH, Mannheim, Germany); and the COLtest®, ADPtest®, ASPItest® and TRAPtest® were used as agonists (Roche, Diagnostics GmbH, Mannheim, Germany); the coefficient of variation for all parameters was ≤15 %.

Blood samples for ROTEM® and Multiplate®Analyzer were immediately transferred to the preheating station of the apparatus. The temperature of the preheating station was adjusted to the chosen analysis temperature. The samples were rested for 30 minutes at the anticipated analysis temperature before initiating the analyses simultaneously at 33 °C and 37 °C, respectively. Clotting time, maximum velocity, time to maximum velocity and maximum cloth firmness were derived from the ROTEM® results. For the Multiplate®Analyzer results, the area under the curve (AUC, AU*min) was used as an indication of platelet aggregation.

Upon admission, on drawing the hypothermic blood sample and the normothermic blood sample, the platelet and white blood cell counts were analysed using an XE-5000 haematology analyser (Sysmex, Kobe, Japan). The international normalised ratio (INR) and fibrinogen (functional) were analysed employing the CS2100i (Sysmex, Kobe, Japan). Blood pH, lactate, and calcium were analysed using ABL800 FLEX (Radiometer, Brønshøj, Denmark).

Samples for the C-reactive protein (CRP) were obtained within 12 hours (90 % within 4 hours) from the blood sample obtained at admission, on hypothermia or on normothermia. CRP was analysed using the Cobas6000 (Roche, Mannheim, Germany).

### Statistics

We chose a minimally relevant difference of 6 seconds in ROTEM®, EXTEM® clotting time, and a 30 AU*min difference in the Multiplate®Analyzer, COLtest® when comparing paired samples obtained at 37 °C with samples obtained at 33 °C during target temperature management. We knew neither the mean nor the standard deviation for patients treated with target temperature management, and we therefore performed the power calculations for ROTEM® analyses based on data from patients with sepsis (EXTEM® mean clotting time of 65 seconds and standard deviation = 10 [15]) and for Multiplate®Analyzer analyses based on healthy individuals (COLtest® mean AUC of 853 AU*min and standard deviation = 53 (data from Rubak et al. [16])). To achieve statistical power of 90 % and a two-sided significance level of 5 %, we had to include 32 patients for the ROTEM® analyses and 35 patients for the Multiplate®Analyzer to identify the chosen minimally relevant difference.

The observations were not normally distributed and therefore data are described as counts, percentages or as medians with ranges. However, inspection of the residuals revealed no deviation from normality, therefore data were analysed using repeated measurements analysis of variance (ANOVA). Using ANOVA, we compared results obtained at 33 °C with results performed at 37 °C. Additionally, to evaluate whether the patient body core temperature interacted with the results, we tested whether the difference between analyses performed at 33 °C and 37 °C in the hypothermic blood sample was equal to the difference between analyses performed at 33 °C and 37 °C in the normothermic blood sample. The temperature used for the analyses and the body core temperatures were categorised. Data from the ANOVA are presented as means with 95 % confidence intervals (CIs).

The Wilcoxon-Mann-Whitney test was used to compare patients treated with 24 hours versus 48 hours of target temperature management in both the hypothermic and the normothermic blood sample. Data were analysed using STATA® version 13 (StataCorp LP, College Station TX, USA).

## Results

Forty patients were included in the study, but five patients had missing ROTEM® or Multiplate®Analyzer values: two patients died, one patient was moved to another facility before the last sample was obtained, and two patients had missing values due to technical error. There were 21 patients randomized to 24 hours of hypothermia and 19 patients to 48 hours of hypothermia.

Among the 40 patients included in the data analyses, 35 patients were male, and the median age was 61 years. The vast majority (93 %) received bystander cardiopulmonary resuscitation and 85 % primarily had a shockable rhythm. Approximately half of the patients were treated with percutaneous coronary intervention. Almost half the patients received ADP inhibitors and one received both ticagrelor and clopidogrel prior to obtaining the hypothermic blood sample. No patients received non-vitamin K oral anticoagulants. The baseline characteristics of the study population are shown in Table 1, 2 and 3.

**Table 1:** Baseline characteristics

Variables	Values
Number of patients, total (%)	40 (100)
Age, years	61 (range 23–79)
Sex, male	35 (88)
Bystander cardiopulmonary resuscitation	37 (93)
Primary rhythm	
Ventricular tachycardia/fibrillation	34 (85)
Asystole, pulseless electrical activity	6 (15)
ROSC^a^, minutes	17.5 (range 5–40)
Coronary angiography	38 (95)
Percutaneous coronary intervention	19 (48)
SAPS II	50 (range 35–75)
Comorbidity, all/*n* (%)	40 (100)
Hypertension	19 (48)
Hyperlipidaemia	13 (33)
Previous acute myocardial infarction	9 (23)
Atrial fibrillation	3 (8)
Diabetes	8 (20)
Chronic obstructive pulmonary diseases/asthma	4 (10)

Characteristics of 40 patients treated with targeted temperature management after cardiac arrest. Results are presented as number of patients (%), or median (range)

*ROSC* return of spontaneous circulation, *SAPS II* Simplified Acute Physiology Score II, *CRP* C-reactive protein

^a^Time from cardiac arrest to ROSC

**Table 2:** Antithrombotic medication

	Prior to cardiac arrest	On hypothermia^a^	On normothermia^a^
Medication, all/*n* (%)	40 (100)	40 (100)	37 (100)
Adenosine diphosphate receptor inhibitor, all:	3 (8)	19 (48)	16 (43)
Ticagrelor	2 (5)	14 (35)	14 (38)
Clopidogrel	1 (3)	6 (15)	2 (5)
Aspirin	15 (38)	29 (73)	25 (68)
Bivalirudin^b^	0 (0)	12 (30)	0 (0)
Low molecular-weight heparin	0 (0)	28 (70)	29 (78)
Unfractionated heparin	0 (0)	19 (48)	0 (0)
Warfarin	3 (8)	0 (0)	1 (3)

Characteristics of 40 patients treated with targeted temperature management after cardiac arrest. Results are presented as number of patients (%), or median (range)

^a^Medication given 24 hours prior to blood sample collection

^b^Use of bivalirudin ended 16–20 hours before obtaining the hypothermic blood sample.

**Table 3:** Conventional coagulation laboratory investigations

Laboratory investigations (normal range)	Upon admission 40 (100)	On hypothermia 40 (100)	On normothermia 37 (100)
Haemoglobin, mmol/l (8.3–10.5)	8.5 (5.7–10.7)	8.1(5.4–10.4)	7 (5.6–9.1)
Platelet count, 10^9/l (145–350)	193 (121–482)	163 (98–399)	142 (57–281)
White blood cell count, 10^9/l (3.5–10.0)	13.4 (4.9–44.6)	9.3 (2.9–22.0)	9.3 (5.2–18.1)
Fibrinogen, functional, μmol/l (5.5–12.0)	6.7 (3.2–12.5)	9.2 (4.5–14.8)	13.3 (7.9–25.9)
International normalised ratio (<1.2)	1.2 (<1– > 10)	1.1 (1.0–4.5)	1.2 (1.1–2.3)
CRP, mg/ml (<8.0)	2.0 (<0.6–76.6)	57.0 (3.1–159.3)	136.7 (10.0–454.0)
Ph (7.37–7.45)	7.26 (7.07–7.43)^a^	7.38 (7.18–7.47)	-
Lactate, mmol/l (0.5–2.5)	2.3 (0.4–12.6)^a^	1.4 (0.6–7.0)	-
Ca^2+^, mmol/l (1.18–1.32)	1.07 (0.94–1.41)^a^	1.17 (1.05–1.59	-

Characteristics of 40 patients treated with targeted temperature management after cardiac arrest. Results are presented as number of patients (%), or median (range)

^a^Upon admission to the intensive care unit

On conventional coagulation laboratory investigations upon admission the majority of haemoglobin and platelet counts were within the normal range, but the white blood cell count was above the normal range. The fibrinogen was within the normal range, but on normothermia the majority of patients had a value above the normal range. The CRP was within the normal range upon admission but increased and was above the normal range in both the hypothermic and the normothermic blood sample. Most patients were still acidotic when they were admitted to the intensive care unit, but pH was within the normal range on hypothermia.

For the hypothermic blood sample, the median body core temperature was 33.1 °C (range 32.6–34.4 °C); for the normothermic blood sample, the median body temperature was 37.5 °C (range 35.8–38.3 °C). The body core temperature was collected in 34 patients (85 %) within 1 hour from collection of the admission blood sample. The median body core temperature at admission was 34.5 °C (range 32.5–37.3 °C).

### ROTEM®

No differences in the ROTEM® results were found between patients using aspirin, low molecular-weight heparin (LMWH) or ADP inhibitors. In three patients using warfarin prior to the cardiac arrest, EXTEM® results performed at 37 °C in the hypothermic blood sample had a prolonged clotting time varying from 96–144 seconds; and time to maximum velocity varied from 154–183 seconds. These results were confirmed by an INR varying from 3.8–4.5 at the same time points in the same patients. The patients on warfarin deviated from the other patients by having a very large difference between EXTEM® clotting time performed at 33 °C compared with 37 °C. The EXTEM® clotting time in these three patients was 32–44 seconds longer in the analyses performed at 33 °C. Thus, patients on warfarin were omitted from the analyses of the difference between results for 33 °C and 37 °C in EXTEM® clotting time and time to maximum velocity.

**Fig. 1:**
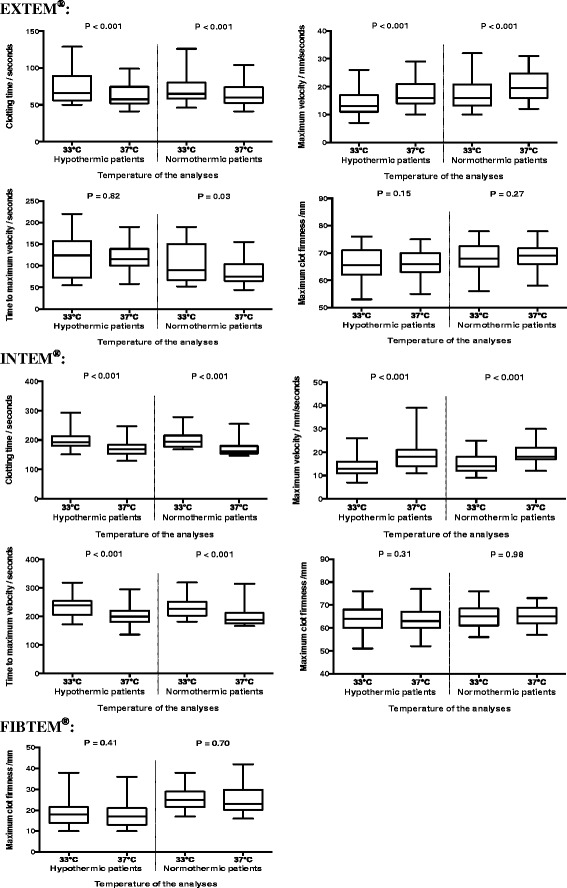
Box plots of ROTEM® results analysed at 33 °C and 37 °C. Forty cardiac arrest patients were included in the study. There were 39 hypothermic samples and 36 normothermic samples included in the ROTEM® analyses. The *p value* is for comparison of results of ROTEM® performed at 33 °C and at 37 °C

The ROTEM® results that were performed at 33 °C and compared with 37 °C, showed an increasing clotting time (all *p* values ≤0.001), decreasing maximum velocity (all *p* values ≤0.001) and an unchanged maximum clot firmness (all *p* values ≥0.15) (Table 4 and Fig. 1). We compared the difference between ROTEM® analyses performed at 33 °C and 37 °C in the hypothermic blood sample with the difference between ROTEM® analyses performed at 33 °C and 37 °C in the normothermic blood sample. We found no significant difference in clotting time, time to maximal velocity, maximum velocity, or maximum clot firmness (all *p* values ≥0.45). However, a significant difference was found in EXTEM® time to maximum velocity (*p* = 0.002).

**Table 4:** The difference in ROTEM® results performed at 33 °C and 37 °C in 40 cardiac arrest patients

	Hypothermia (body core temp median 33.1 °C), *n* = 39 ROTEM® 33 °C versus 37 °C		Normothermia (body core temp median 37.5 °C), *n* = 36 ROTEM® 33 °C versus 37 °C	
	Mean difference (95 % CI)	*P* value	Mean difference (95 % CI)	*P* value
EXTEM®				
Clotting time, sec	8 (4; 12)	<0.001	9 (5; 13)	<0.001
Maximum velocity, mm/sec	–4 (–4; -3)	<0.001	–4 (–5; –3)	<0.001
Time to maximum velocity, sec	2 (–12; 15)	0.82	17 (2; 33)	0.03
Maximum clot firmness, mm	0 (–1; 0)	0.15	0 (–1; 0)	0.27
INTEM®				
Clotting time, sec	27 (20; 34)	<0.001	28 (20;36)	<0.001
Maximum velocity, mm/sec	–5 (–6; –4)	<0.001	–4 (–5; –4)	<0.001
Time to maximum velocity, sec	32 (19; 45)	<0.001	33 (19; 47)	<0.001
Maximum clot firmness, mm	0 (–1; 0)	0.31	0 (–1; 1)	0.98
FIBTEM®				
Maximum clot firmness, mm	1 (–1; 3)	0.41	0 (–2; 2)	0.70

*temp* temperature, *n* number, *CI* confidence interval

The HEPTEM® results were similar to the INTEM® results; this was supported by an INTEM®/HEPTEM® clotting time ratio of 1.0.

### Multiplate®Analyzer

**Fig. 2:**
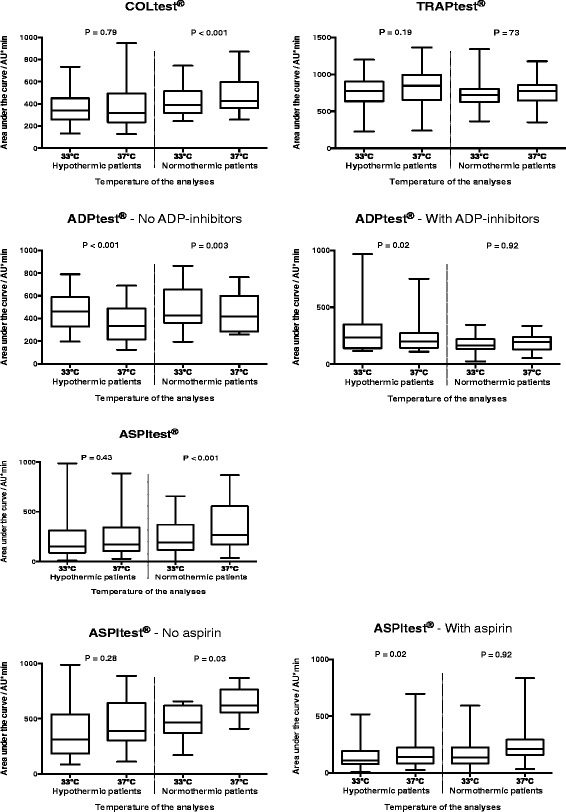
Box plots of Multiplate®Analyzer results analysed at 33 °C and 37 °C. Forty cardiac arrest patients were included in the study. There were 39 hypothermic blood samples and 36 normothermic blood samples included in the Multiplate®Analyzer analyses. The *p* value is for comparison of the results performed at 33 °C and at 37 °C

The difference between Multiplate®Analyzer analyses performed at 33 °C and 37 °C in the hypothermic blood sample were significantly different from the difference between Multiplate®Analyzer analyses performed at 33 °C and 37 °C in the normothermic blood sample in the COLtest® (*p* = 0.05) and the ASPItest® (*p* = 0.02): there were no differences in either test in the Multiplate®Analyzer results performed at 33 °C and 37 °C when the patients were hypothermic (COLtest® *p* = 0.79; ASPItest® *p* = 0.43); however, in the normothermic patients, there was decreased aggregation at 33 °C (COLtest® *p* < 0.001, ASPItest® *p* < 0.001) (Table 5 and Fig. 2).

**Table 5:** The difference in Multiplate®Analyzer measurements performed at 33 °C and 37 °C in 40 cardiac arrest patients

	Hypothermia (body core temp median 33.1 °C), *n* = 39 33 °C versus 37 °C		Normothermia (body core temp median 37.5 °C), *n* = 37 33 °C versus 37 °C	
	Multiplate®Analyzer, (AU*min)		Multiplate®Analyzer, (AU*min)	
	Mean difference (95 % CI)	*P* value	Mean difference (95 % CI)	*P* value
COLtest®^a^	–4 (–30; 23)	0.79	–62.0 (–87; –37)	<0.001
TRAPtest®	–38 (–96; 20)	0.19	–12.5 (–84; 59)	0.73
ASPItest®				
All^a^	–24 (–83; 35)	0.43	–106 (–158; –53)	<0.001
No aspirin (*n* = 9)	–71 (–199; 57)	0.28	–176 (–336; –16)	0.03
Aspirin (*n* = 26)	–27 (–53; –1)	0.04	–76 (–108; –45)	<0.001
ADPtest®				
No ADP inhibitors (*n* = 20)	106 (68; 144)	<0.001	51 (18; 83)	0.003
ADP inhibitors (*n* = 18)	38 (6; 69)	0.02	2 (–33; 37)	0.92

*temp* temperature, *n* number, *CI* confidence interval, *ADP inhibitors* adenosine diphosphate receptor inhibitors, AU*min (y-axis is expressed in Aggregation units (AU) and x-axis in minutes). ^a^The body core temperature significantly affected the differences in results at 33 °C and 37 °C

On the ADPtest® there was increased aggregation when analysed at 33 °C compared with 37 °C in hypothermic patients, and in normothermic patients who were not given ADP inhibitors (all *p* values <0.02). The TRAPtest® was not dependent on the temperature of the analyses or the body core temperature (all *p* values ≥0.18) (Table 5 and Fig. 2).

### Duration of hypothermia

In the hypothermic blood sample, the EXTEM® maximum velocity was longer (*p* = 0.02) in the 48-hour group. In the normothermic blood sample, we found a longer INTEM® clotting time and time to maximum velocity (all *p* < 0.02) in the 48-hour group. Besides these differences, the ROTEM® and Multiplate®Analyzer results were comparable in the two intervention groups in both the hypothermic and the normothermic blood samples.

## Discussion

We conducted a prospective study with paired observations on hypothermia and subsequent normothermia to investigate whether ROTEM® and Multiplate®Analyzer results were temperature-dependent. With ROTEM® there was compromised initiation of coagulation when analysis was performed at 33 °C, but no differences in maximum clot firmness. The ROTEM® results did not appear sensitive to the patient’s body core temperature. The Multiplate®Analyzer measurements were influenced by the temperature of the analyses and by the patient’s body core temperature, but in different ways depending on the agonist used.

Previous studies have investigated whether the temperature of the analyses influenced the thromboelastometric measurements. However, these studies included only small sample sizes and were carried out in vitro in samples from normothermic healthy individuals [17–19] or in hypothermic patients [20–22]. Our ROTEM® results are in accordance with those reported in these previous, smaller studies, and they indicate prolonged clotting initiation and no difference in clot strength when performed at 33 °C, and compared with 37 °C. To our knowledge, no previous studies have examined whether this impairment is reduced when the patients are rewarmed. We therefore investigated whether the difference between analyses performed at 33 °C and 37 °C would be the same for hypothermic and normothermic patients.

We found that patients using warfarin had a very long clot formation time, which is consistent with INR above the usual therapeutic range. Notably, in these patients treated with warfarin we observed a very large difference in EXTEM® clotting time between analyses performed at 33 °C and 37 °C. The present study included only patients with no pre-existing bleeding disorders; as such, we do not know whether there would be a large difference between analyses performed at 33 °C and 37 °C in patients with pre-existing impaired haemostasis, as was seen in patients treated with warfarin.

The Multiplate®Analyzer was used in three previous studies to investigate the ability of platelets to aggregate during hypothermia [23–25]. Two of these studies were performed in vitro [23, 24], and the third investigated the difference between haemostasis in hypothermia and in subsequent normothermia [25]. All three studies were small and there was no difference between analyses performed at 33 °C and at 37 °C, except in the study by Kander et al. who demonstrated a significant difference using the ASPItest® in normothermic patients [25], which is in accordance with our ASPItest® results. However, our ADPtest® and COLtest® results differed from the results reported in previous studies, probably because the previous studies were underpowered due to the large standard deviation in the Multiplate®Analyzer results.

We observed that platelet aggregation sensitivity to hypothermia had different patterns dependingt on the agonist used; however, the reason for this remains unresolved. We observed decreased platelet aggregation using the COLtest® and the ASPItest® when analysing normothermic blood at 33 °C. However, there was more aggregation at 33 °C using the ADPtest®. There was also increased platelet aggregation in other studies when platelets were stimulated with ADP on hypothermia [26, 27]. Whether there is increased or decreased platelet aggregation during target temperature management is intensely debated, especially in patients with a risk of stent thrombosis after percutaneous coronary intervention [28–31].

The COLtest® and ASPItest® results were not affected in the hypothermic blood sample, but we observed a difference in the normothermic blood samples. Thus, targeted temperature management might inhibit the COLtest® and ASPItest®, but rewarming the blood sample for 30 minutes at the preheating station did not terminate the inhibition.

In clinical practice, ROTEM® and Multiplate®Analyzer analyses are performed at 37 °C. However, by measuring the results at 37 °C instead of 33 °C in patients treated with targeted temperature management, we overlooked a small compromised initiation and propagation of the coagulation using ROTEM®. With the Multiplate®Analyzer we overlooked a small increased aggregation using the ADPtest®. It is doubtful, however, whether these rather small differences in ROTEM® and Multiplate®Analyzer have any clinical impact. Therefore, we recommend maintaining the practice of analysing whole blood coagulation tests at 37 °C, irrespective of whether the patient’s body core temperature is 33 °C or 37 °C.

The strength of this cohort study is that it involves a large sample size with paired data obtained on both hypothermia and subsequent normothermia. The study included patients with cardiac arrest, which increased the external validity for study of critically ill patients. Moreover, there was a small time frame for each sampling and the each blood sample was analysed simultaneously. The advantage of ROTEM® compared to plasma-based conventional coagulation tests, such as APTT and INR, is that ROTEM® is performed in whole blood and thereby, is superior for reflection of in vivo coagulation. ROTEM® results are performed quickly, and the results are more suitable for targeted haemostatic treatment in bleeding patients [32]. However, ROTEM® has a limited capability to detect antithrombotic treatment, and as platelet function is not reflected in the ROTEM® analysis, it is important to supplement it with platelet aggregation tests such as the Multiplate®Analyzer. The agonists used in the present study were sensitive to the use of ADP inhibitors and acetysalicylic acid [13]. Hence, the use of both these two instruments increased the capability to investigate the entire coagulation. However, none of the included methods reflect the entire haemostatic process, as this also includes vasoconstriction, endothelium function, and significance of flow. Moreover, the relatively high imprecision of these methods must be taken into consideration when interpreting the results.

We tested whether the difference between samples analysed at 33 °C and 37 °C were affected by the patient being hypothemic or normothermic. Thus, the timeframe between the hypothermic and the normothermic blood sample meant that confounding was possible. Potentially confounders were the “post cardiac arrest syndrome” and the reheating of the patient, which causes inflammation and thereby, might stimulate coagulation [33]. Suspicion of increased inflammation between the hypothermic and the normothermic blood sample is reinforced by increase in fibrinogen and CRP. However, if the ROTEM® and Multiplate®Analyzer results were substantially influenced by inflammation, the results from the present study would only be affected if inflammation affected analyses performed at 33 °C and 37 °C unequally, but notably, this is not likely the case. The use of unfractionated heparin or bivalirudin administered in relation to the percutaneous coronary intervention could probably be neglected owing to the long time frame between the administration of the drugs and the blood sampling time.

The patients were treated with two different interventions, namely 24 hours and 48 hours of targeted temperature management. Comparing the ROTEM® and Multiplate®Analyzer results in the two intervention groups, we found deviating, non-conclusive signs of compromised haemostasis in INTEM® in the 48-hour group. If we presume that prolonged targeted temperature management influenced the 48-hour group, then there is a small risk that we might have overlooked the impact of body core temperature on the ROTEM® data analyses. Apart from this small difference, ROTEM® and Multiplate®Analyzer results were comparable. We therefore found it reasonable to combine the two interventions groups into one.

## Conclusions

In conclusion, the present study suggested that ROTEM® results are not sensitive to the patient’s body core temperature. There was a small compromised initiation of haemostasis when the analysis was performed at 33 °C, but no difference in clot strength. It is questionable whether this small difference is of clinical relevance. The analyses of platelet aggregation were influenced by hypothermia in different ways depending on the agonist used and on whether patients were hypothermic or normothermic.

Analysis of whole blood coagulation tests should be performed at a temperature of 37 °C, irrespective of whether the patient’s body core temperature is 33 °C or 37 °C.

## Key messages

- Thromboelastometry and platelet aggregation tests are used to evaluate bleeding patients, but the analyses are performed at 37 °C, irrespective of the patient’s body core temperature
- Thromboelastometry showed slightly compromised initiation of haemostasis when analysed at 33 °C compared with 37 °C, but no difference in clot strength
- The platelet aggregation test was dependent on both the temperature of the analyses and the body core temperature
- The temperature of whole blood coagulation tests should be kept at 37 °C, irrespective of whether the patient’s body core temperature is 33 °C or 37 °C

## Abbreviations

ADP inhibitors: adenosine diphosphate receptor inhibitors
ANOVA: analysis of variance
AU: Aggregation units
AUC: area under the curve
CI: confidence intervals
CRP: C-reactive protein
INR: international normalised ratio
LMWH: low molecular weight heparin
ROSC: return of spontaneous circulation
